# Quantification of radioactivity by planar gamma-camera images, a promoted method of absorbed dose in the thyroid after iodine-131 treatment

**DOI:** 10.1038/s41598-018-28571-y

**Published:** 2018-07-05

**Authors:** Yuhao Li, Huawei Cai, Guohua Shen, Fuwen Pang, Ping Dong, Lin Li

**Affiliations:** Department of nuclear medicine, West China Hospital of Sichuan University, 37, Guoxue lane, Wuhou District, Chengdu, Sichuan province P. R. China

## Abstract

Iodine-131 (^131^I) is an essential and widely used radioisotope in thyroid diseases and animal experiments. Planar imaging has been considered the most popular method for ^131^I thyroid uptake radioactive activity quantification. The ROI defining section is essential and can affect the accuracy of quantitative results. However, a consistent method has not been proposed. In this study, a UC-ROI defining method based on ULWL setting and colour display grade was applied. Three steps were performed: image acquisition of five standard activity models and obtaining the exact value that the counts per radioactive activity contributes to the ROI; image acquisition of 20 rat thyroids and obtaining the counts of the ROI (thyroid); and calculating the rat thyroid radioactive activity and comparing these values with the true values. The accuracy of quantification activity of ^131^I in rat thyroid reached 2.62% ± 0.41%. The mean quantification within 5% could be achieved in 19 of 20 rat thyroids. No significant difference existed between calculated thyroid ^131^I activity and true values with a paired matched-test (t = −0.384, P = 0.706 > 0.05). The results indicated that with the UC-ROI defining method, more accurate thyroid uptake ^131^I radioactive activity quantification by SPECT planar imaging can be achieved *in vivo* rat study.

## Introduction

With the increasing use of radiopharmaceuticals both in clinics and animal experiments, precise estimation of the absorbed dose in patients is crucially required to evaluate the prognosis and side effects^1^. Planar images of a gamma camera were used for activity estimated several decades previously^2,3^; however, the deviation between estimated activity and true activity could be up to 10% or more for certain organs^4–6^, which was mainly caused by region of interest (ROI) definition, background, scatter and attenuation^1,4,7^. In recent decades, by the development of fused imaging technology of single photon emission computed tomography and computed tomography (SPECT/CT), attenuation correction, scatter correction and partial volume correction, absolute organ activity quantification has reached a new level. Using the method of quantitative SPECT/CT, the accuracy has increased and in a manner similar to positron emission computed tomography (PET), even the standard uptake value (SUV) used for diagnosis can be obtained^8,9^. However, because of the complexity to be achieved, quantitative SPECT/CT is rarely applied in clinical practice. For clinical practice and animal researches, a simple, accurate, easy-to-reach quantitative method of planar imaging is desired.

Iodine-131 (^131^I) is an essential and widely used radioisotope in thyroid diseases, and planar imaging has been considered the most popular method for ^131^I thyroid uptake radioactive activity quantification^5,10–12^. The sensitivity known as count rate per unit activity or calibration factor must be known in order to convert the acquired count rate to activity^5^. To obtain sensitivity data, pamphlet 16 of the Medical Internal Radiation Dosimetry committee (MIRD) recommend an independent measurement of a standard, with known activity, within a standardized geometry in air relative to the scintillation camera, using designed gamma camera acquisition parameter settings. The ^131^I radioactive activity of thyroid uptake is calculated using the equation ((thyroid counts − background counts)/(sensitivity × time))^5,12,13^. The thyroid counts and background counts are obtained by drawing an ROI of the target tissue and an ROI of the background region. For quantification of planar imaging, defining an ROI is an essential procedure that can affect the accuracy of the quantitative results. However, studies of thyroid radioactive activity quantification did not provide a consistent method for defining the ROI. Most researchers defined the ROI according to visual characteristics and drew a region containing the thyroid^10,12^, but the visual character is a fuzzy concept. Additionally, several researchers adopted other methods for defining the ROI of the target tissue, such as choosing an 85% isocount contour of the thyroid gland^13^ or considering system spatial resolution (FWHM)^5^. In a word, the criterion for defining the ROI of the target tissue is unified, and studies are rarely designed to use a normative, accurate and simple ROI defining method.

Recently, with the development of image data processing workstations, a more accurate ROI and background regions can be defined, which has made the exact quantification of planar imaging possible. In this study, we adopted a new method for defining the ROI border based on the upper limit window level (ULWL) and a ring-like shape of the background to obtain corrected ROI counts and achieve quantification of thyroid uptake ^131^I activity. An alternative conventional ROI defining method by visual characteristics was applied as a reference. Models with measured ^131^I activity and rats injected with ^131^I were used to verify accuracy. Three steps were performed to derive absolute values for radioactive activity in Bq (1 µCi = 3.7 × 10^4^ Bq) from planar images and verify accuracy^5,13^: image acquisition of standard activity models and obtaining the exact value that the counts per radioactive activity contribute to the ROI; image acquisition of rat thyroids and obtaining the counts of the ROI (thyroid); and calculating the rat thyroid radioactive activity and comparing these values with the true values. We tried to verify the accuracy of the quantification in rats experiments.

## Materials and Methods

### Image Acquisition of Standard Activity Models

A plastic disc (diameter, 60 mm; height, 15 mm) covered with a layer of tri-distilled water (depth, 5-mm approximately) was used as the model. A 5-mm depth water is used for simulating the thyroid underneath the skin. The total dose of ^131^I activity was measured with a radioactivity meter (RM905a, Beijing Hicheer Sci-Tec Co., Ltd.) before being introduced into the model with a syringe. After repeated washing with the tri-distilled water in the model, the syringe needed another measurement for residual radioactive activity. The difference between the two measurements was regarded as the true value of radioactive activity remaining in the model.

A high energy general purpose (HGCP) collimator from a SPECT/CT was used to acquire planar images (GE Discovery, NM/CT 670). One collimator was proposed since only the anterior image was required. The scan was undertaken for 200 seconds with the energy window at 10%, centred on 364 KeV. The matrix was settled as 256 × 256, and no zoom factor was used. The standard models were measured at a distance of 10-cm from the HGCP collimator. A 10-cm distance was chosen because it was widely used in clinical practice and suitable for all patients, especially women with large breasts^12^.

We designed five kinds of radioactive activity models, 3.7 × 10^6^ Bq (100 µCi) for Model 1 (M_1_), 7.4 × 10^6^ Bq (200 µCi) for Model 2 (M_2_), 1.48 × 10^7^ Bq (400 µCi) for Model 3 (M_3_), 2.96 × 10^7^ Bq (800 µCi) for Model 4 (M_4_), and 5.92 × 10^7^ Bq (1600 µCi) for Model 5 (M_5_). Images for each activity model were collected five times to avoid statistical fluctuation and measuring error.

Additionally, each image acquisition lasted for 200 seconds, which was much shorter compared with the radioactive half-time of ^131^I (approximately 8 days); therefore, we felt that the activity remained unchanged during acquisition.

### Image Acquisition of Rat Thyroids

Twenty rats (weight, 290–310 g; Sprague Dawley; male) were studied for estimating thyroid ^131^I activity and evaluating accuracy. For each rat, a random amount of ^131^I activity, ranging from 1.11 × 10^7^ Bq to 1.11 × 10^8^ Bq (300 µCi to 3 mCi), was introduced through intraperitoneal injection. The images were collected approximately 6–8 hours after injection.

The imaging system and parameters of rat thyroids should be maintained with standard radioactive activity models; otherwise, the data of rat thyroid images and standard model images would not be comparable.

After imaging, the rats were sacrificed by isoflurane overdose immediately. The thyroids were dissected and measured in the radioactivity meter (RM905a, Beijing Hicheer Sci-Tec Co., Ltd.) to obtain the true activity values as the gold standard.

Additionally, for each rat, it took nearly 20 minutes from the beginning of imaging to the accomplishment of extracted thyroid activity measurement. The time was much shorter compared with the ^131^I half-time. For this reason, we regarded the value of rat thyroid uptake activity as a constant.

### Method of ROI Selection Control

Traditionally, the ROI is drawn according to the visual characteristics, and many studies have been based on this method^10,12^. However, what has been ignored is that the visual characteristics are affected by the upper limit of the window level (ULWL) setting, which can affect the visual characteristics.

**Figure 1:**
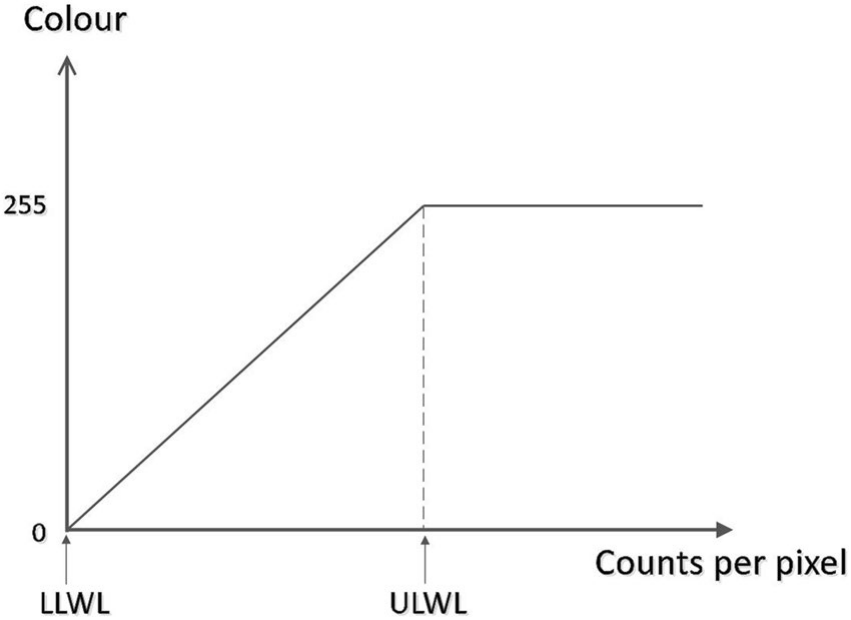
Relationship between colour brightness and windows level. The change in colour brightness maintains a linear relationship with the counts per pixel within the range from LLWL to ULWL. For the pixels with larger counts than ULWL, the colour display grade remains the brightest.

**Figure 2:**
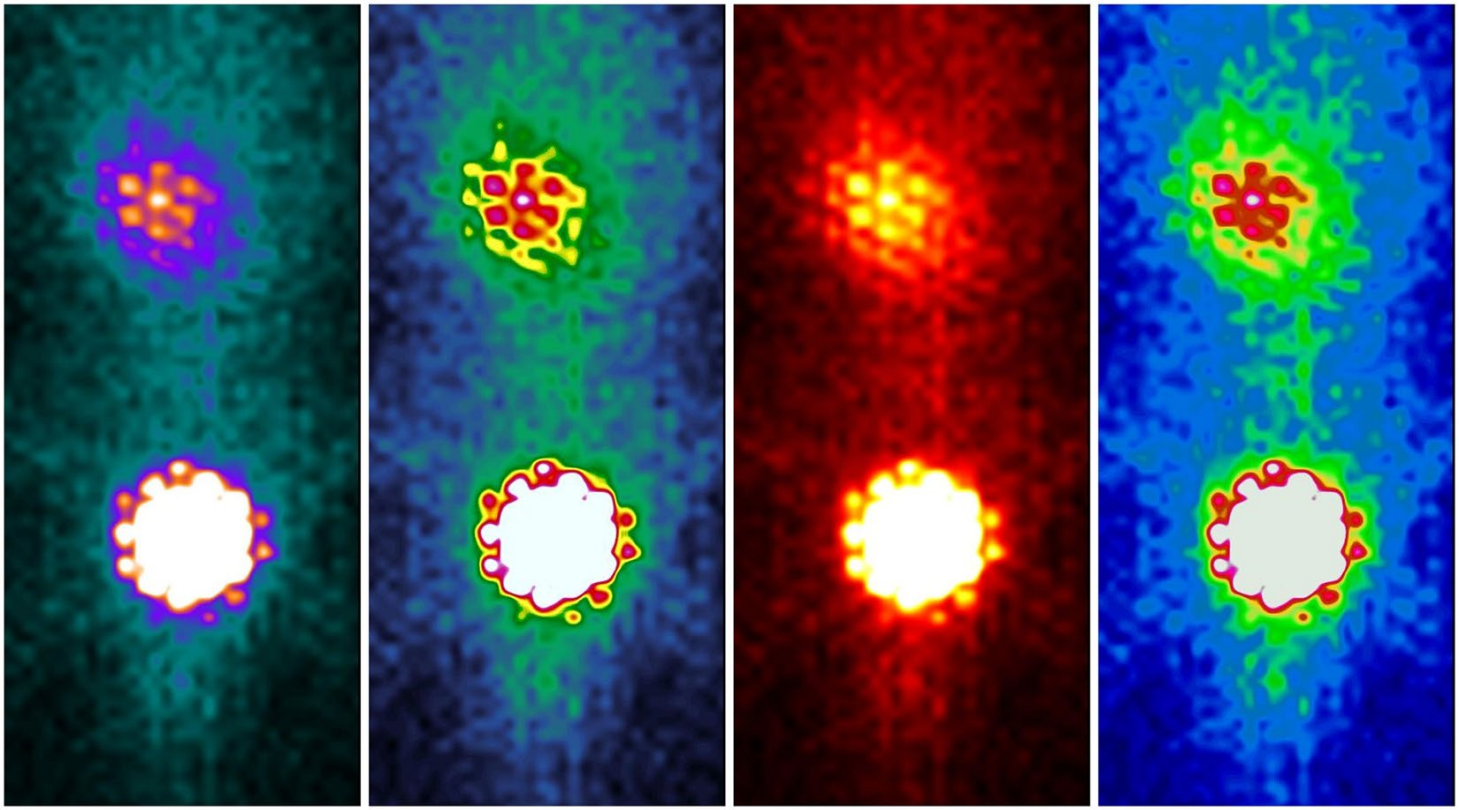
Four kinds of colour display patterns. The colour display patterns for examples, left to right-GE colour, French, Hot Iron, and XT1 Rainbow. The shade of colour represents the counts per pixel, and the counter lines of the colour display grade can be observed in different patterns. It is the counter lines that are used for defining ROI borders visually. The colour display pattern of GE colour was used in this research.

The relation between visual characteristics and ULWL is illustrated in Fig. 1 (copied from GE Healthcare, Direction 5336813-1ZH-CN Revision 2, Processing and Review System, User Guide). The horizontal axis represents the counts per pixel and the vertical axis represents the grade of colour display. The value of counts per pixel varies from 0 to the maximum according to the planar image. The grade of colour display improves from 0 to 255 (from the darkest to the brightest) in correspondence with the value of counts per pixel changing from the lower limit window level (LLWL) to the ULWL. The pixels with the same counts are shown in the same grade of colour display, similar to a contour line (Fig. 2 shows several colour display patterns and, in this research, the GE colour pattern was used). For the pixels that had higher counts than the stated ULWL, the colour display was the brightest.

To achieve an accurate ROI region, a proper ULWL should be adopted. For each planar image, both the standard models and rat thyroids, the value of ULWL was set according to the following equations:

1 $${\rm{\alpha }}=\frac{t}{p}$$

2 $${\rm{ULWL}}={\rm{\mu }}\times {\rm{\alpha }}$$

*t* represents total counts of the whole planar image*p* represents the amount of pixelsμ is a constant (equals to 50 in this study).

The sensitivity (count rate per unit activity) for a particular gamma camera (SPECT collimator) is stable, which means radioactive sources with larger radioactive activity will lead to a larger total counts in the planar image over the same period of time; in other words, *t* is affected by the amount of radioactive activity. The *p* is settled as a constant for an exact planar imaging depending on the matrix area. In this research, a matrix of 256 × 256 was applied for all the planar image acquisitions, so the *p* was equal to 65536.

**Figure 3:**
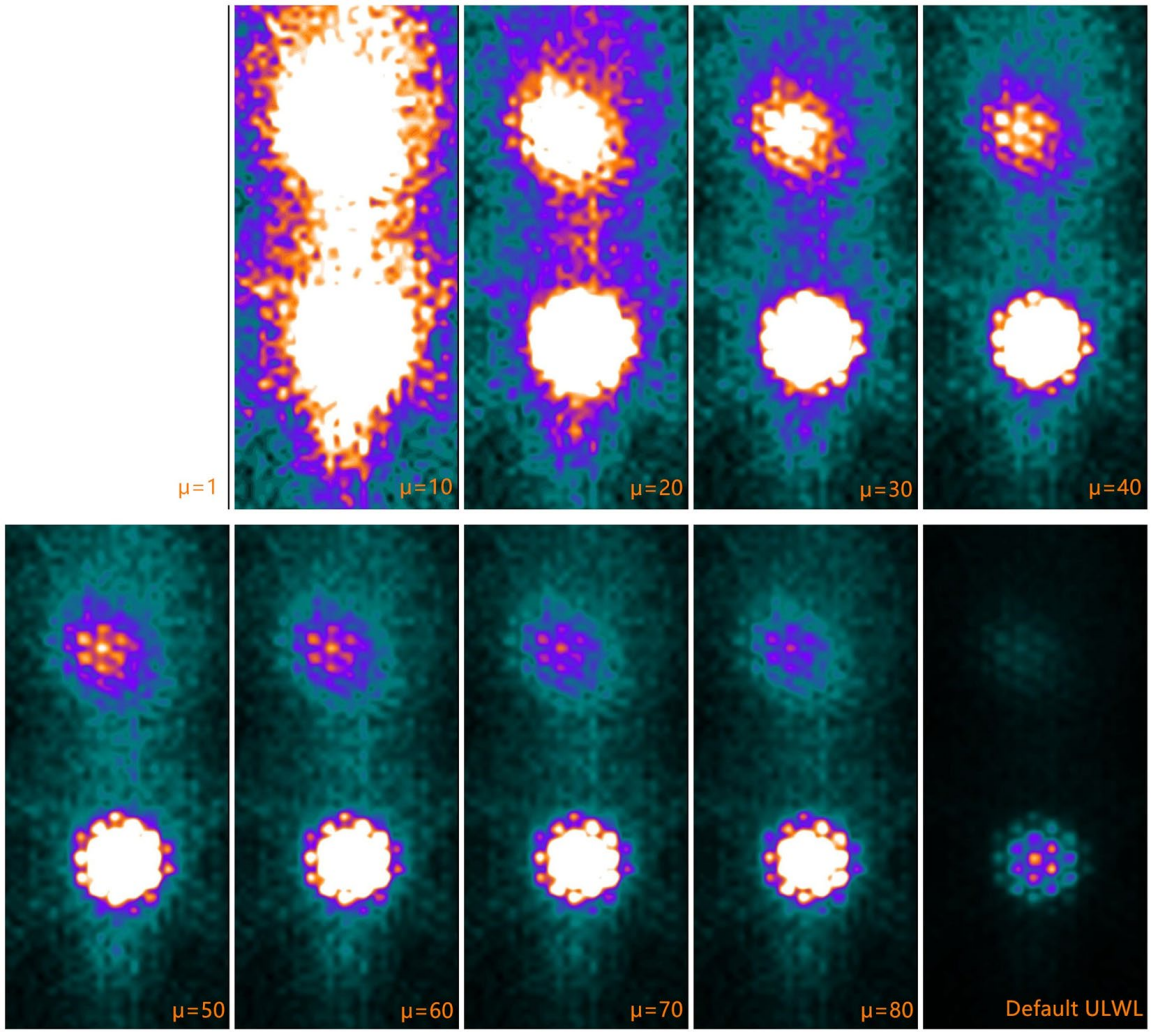
Planar images with different μ values of the same radioactive source (a rat). We tested μ with different values (from 1 to 80) and obtained various results for the images (the μ for default ULWL is 366). A suitable value of μ must satisfy the requirement that the colour display border of the target tissue should be recognized. In this research, a value of 50 for μ was adopted (other values such as 40, 60, and 70 will also be reliable; the key point is that the value of μ should stay the same for all data processing and ROIs defining).

The coefficient μ is a constant that can be set from 0 to 100 or more. The planar images with different μ values of the same radioactive source are shown in Fig. 3. The default system value for ULWL is equal to the maximum count per pixel. We can determine that it is not suitable if a small μ is applied because the border of the ROI region is not clear. In this research, we chose μ as 50 because the planar images had a better visual characteristic at this condition (additionally, other values such as 40 and 60 will also be fine). Once the μ is decided, each planar image should take the one and only coefficient for setting the ULWL and obtaining the ROI.

**Figure 4:**
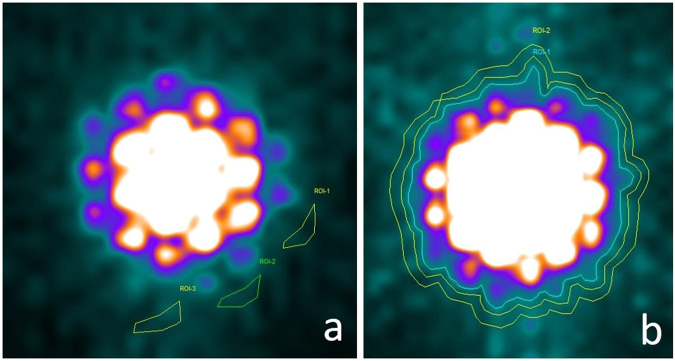
Method of ROI selection. Three background ROIs were drawn according to the traditional method in (**a**). The average counts per pixel for ROI-1, ROI-2, and ROI-3 are 18.143, 21.714 and 30.500, respectively. Distinct differences could be noticed and these differences would result in inaccuracy for radioactive activity quantification. In (**b**) the ROI-1 in blue colour represents the target tissue (rat thyroid) defined according to the UC-ROI drawing method. The ROI-2 in yellow colour, which is drawn in a narrow circle shape next to ROI-1, represents the background region.

After the ULWL is set, a counter line of colour grade should be chosen for drawing the ROI border. The specific colour grade used for defining the ROI border should be maintained in all of the planar image processing. In our study, a colour display pattern named GE colour was used and a specific colour grade was adopted for drawing an ROI border in the manner of ROI-1 in Fig. 4b.

Since this method is based on ULWL setting and colour display grade, we will use the UC-ROI drawing method for short in the following. After drawing ROI, the total counts of the ROI and average counts per pixel of the ROI can be obtained from the imaging workstation.

### Method of Background ROI Selection

Traditionally, the background ROI is drawn near the thyroid and is always defined over an area caudal and lateral in relation to that of the target tissue ROI^5,10^. However, as is illustrated in Fig. 4a, there are differences among the average background per pixel for different ROIs. Accurate quantification will not be achieved if the differences in background ROI selection remain. Therefore, we carried out a new method for background selection, i.e., a ring-like background ROI drawing method. As is shown in Fig. 4b, a narrow annular background region is drawn next to the target tissue ROI. The differences in counts per pixel around the target tissue ROI are averaged and eliminated by this method. Moreover, this method is easy to follow, and standardizing the background ROI selection will benefit the repeatability and reliability of results.

After the background ROI is selected, the average counts per pixel can be read from the imaging workstation.

### Acquisition of calculated radioactive activity for rat thyroid

Once the total counts of ROI, the average counts per pixel of the ROI for the target tissue and the average counts per pixel for the background region are obtained, the corrected total counts for the target tissue ROI (δ) can be calculated as follows:

3 $${\rm{\delta }}={\rm{S}}\times \frac{{{\rm{V}}}_{1}-{{\rm{V}}}_{2}}{{{\rm{V}}}_{1}}$$

S represents total counts of the ROI for the target tissueV_1_ represents the average counts per pixel of the ROI for the target tissueV_2_ represents the average counts per pixel for the background region.

For standard models, δ_1_, δ_2_, δ_3_, δ_4_ and δ_5_ represent the corrected total counts for M_1_, M_2_, M_3_, M_4_ and M_5,_ respectively. A_1_, A_2_, A_3_, A_4_ and A_5_ represent the exact radioactive activity measured by the radioactive meter for each model. The standard counts per radioactive activity (τ) contributing to δ can be acquired by the following equation:

4 $$\begin{array}{rcl}{{\rm{\tau }}}_{{\rm{n}}} & = & \frac{{{\rm{\delta }}}_{{\rm{n}}}}{{{\rm{A}}}_{{\rm{n}}}}\\ {\rm{\tau }}^{\prime}  & = & \frac{1}{5}\times \sum {{\rm{\tau }}}_{{\rm{n}}}\end{array}$$

τ_n_ is the calculated standard counts per radioactive activity contributing to each model (M_1_ ~ M_5_), and τ′ is the average of τ_n_.

The radioactive activities of rat thyroids are calculated as follows:

5 $${\rm{A}}^{\prime} =\frac{{\rm{\delta }}^{\prime} }{{\rm{\tau }}^{\prime} }$$

A′ represents the estimated radioactive activity of the rat thyroid, unit of 3.7 × 10^4^ Bq (1 µCi). δ′ represents the corrected total counts for the ROI of the rat thyroid.

After every A′ collected, the accuracy can be verified by a comparison of A′ and true radioactive activity of the rat thyroid measured by a radioactivity meter.

### Conventional quantitative method by visual characteristics

ROI defining method by visual characteristics was applied as a reference. The calculating procedure (equations 3–5) kept consistent with UC-ROI defining method. But the methods for ROI selection and background ROI selection were different. For the reference, ROI was selected directly on the planar imaging of default ULWL by visual characteristics, without any adjustment of the ULWL value. And background ROI was selected according to the method shown in Fig. 4a.

### Data processing and statistical analysis

All the image data were processed at the Xeleris Functional Imaging Workstation (GE, Xeleris version 3.1). SPSS software (Version No. SPSS Statistics 22.0 version) was used for statistical processing. The numerical data were reported as means ± standard error (S.E.). A *P* value less than 0.05 was considered significantly different.

### Ethical approval and consent to participate

Approved by West China Hospital of Sichuan University Biomedical Research Ethics Committee (Number: IACUC-2016029a). All experiments were performed in accordance with relevant guidelines and regulations. This article does not contain any studies with human participants performed by any of the authors.

### Availability of data and material

Please contact author for data requests (including images).

### Data availability

All the data including the Excel database and the DICOM data are available.

## Results

### Results of the Standard Activity Model

**Figure 5:**
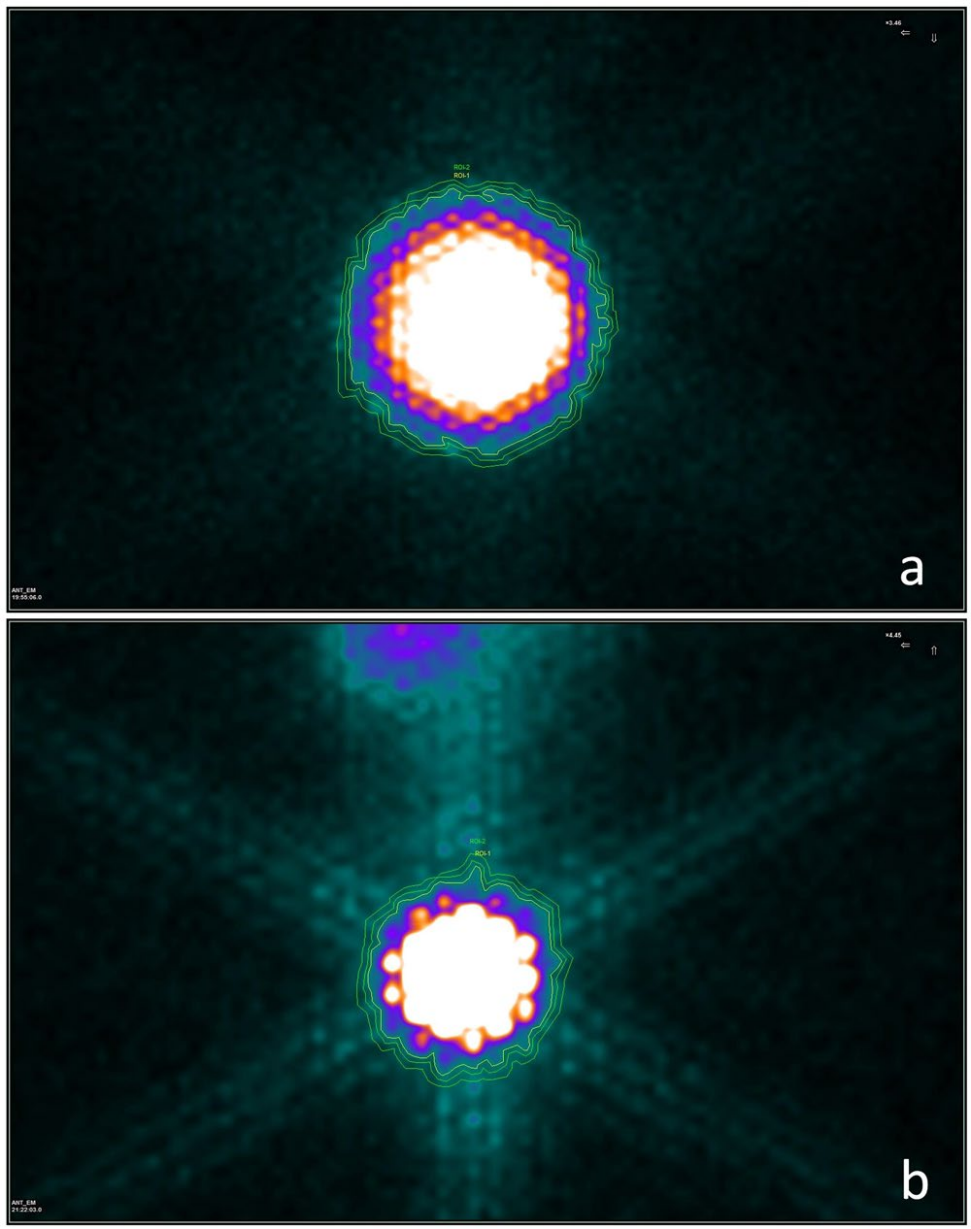
Examples for ROI selection of standard model and rat thyroid. (**a**) Shows an example of processing of the standard model. The default ULWL is 838. According to equations (1) and (2), we adjusted the value of ULWL to 454 (μ = 50 was adopted). Then, the ROI-1 for the target tissue and ROI-2 for the background were selected according to the UC-ROI defining method and the ring-like background defining method. (**b**) Is a planar image of a rat thyroid. The processing method was maintained with (**a**) and we adjusted the ULWL from the default value (3701) to 416 (μ = 50).

All the images of five standard activity models were processed by drawing a UC-ROI and a circle background ROI (Fig. 5a). Because of manual operation and the syringe remaining, the actual activities of the five models were 3.589 × 10^6^ Bq (97 µCi), 6.808 × 10^6^ Bq (184 µCi), 1.3875 × 10^7^ Bq (375 µCi), 2.9896 × 10^7^ Bq (808 µCi) and 5.621 × 10^7^ Bq (1533 µCi), with small differences compared with the designed values of standard activities. However, this would not affect the consequences.

The values of τ_n_ for each model were 159.6, 161.8, 161.1, 159.8 and 165.7, and the average τ′ was 161.6. There was good consistency among τ_n_ for different models with various radioactive activities (standard deviation, 2.467). The exact counts per radioactive activity (per 3.7 × 10^4^ Bq, 1 µCi) contributing to the target tissue was ascertained as 161.6, with a fixed 200 s, 10-cm planar SPECT imaging acquisition, a UC-ROI drawing method and a ring-like background region selection.

### Activity Quantification of ^131^I in Rat Thyroid

Twenty planar images of rats were acquired. According to the UC-ROI drawing method, the value of ULWL for each image was settled and the ROI of the target tissue was defined based on the same visual characteristics. All the values of S and V_1_ for each rat thyroid ^131^I image were collected. Then, a circle background region was drawn next to the UC-ROI border to obtain the value of V_2_ (Fig. 5b).

The results of rat thyroid ^131^I activity quantification are summarized in Table 1, including ULWL, δ′, calculated thyroid ^131^I activity (A′), true thyroid ^131^I activity and the deviation. The average for deviations of the calculated activity from the gold standard values was 0.69% ± 0.71%. The average for absolute deviations of calculated activity from the gold standard values was 2.62% ± 0.41%. The mean quantification within 5% could be achieved in 19 of 20 rat thyroids. No significant difference existed between calculated thyroid ^131^I activity and gold standard values with a paired matched-test (t = −0.384, P = 0.706 > 0.05).

**Table 1:** Results of rat thyroid 131I activity quantification.

Rat Number	ULWL	δ′	A′(=δ′/τ′) (µCi)	True activity measured in radioactivity meter (µCi)	Deviation from true value (%)	Absolute value
1	314	52022.8	321.92	307	4.86%	4.86%
2	75	22723.6	140.62	131	7.34%	7.34%
3	73	18780.0	116.21	111	4.69%	4.69%
4	230	70225.9	434.57	436	−0.33%	0.33%
5	481	147722.9	914.13	945	−3.27%	3.27%
6	462	144833.4	896.25	918	−2.37%	2.37%
7	263	47358.9	293.06	305	−3.91%	3.91%
8	264	48684.9	301.27	312	−3.44%	3.44%
9	174	54866.7	339.52	345	−1.59%	1.59%
10	52	7959.9	49.26	50	−1.48%	1.48%
11	58	14505.9	89.76	88	2.00%	2.00%
12	58	8028.4	49.68	51	−2.59%	2.59%
13	57	13924.4	86.17	83	3.82%	3.82%
14	416	149851.7	927.30	930	−0.29%	0.29%
15	356	107716.3	666.56	656	1.61%	1.61%
16	391	148907.6	921.46	918	0.38%	0.38%
17	348	131567.9	814.16	813	0.14%	0.14%
18	169	40738.8	252.10	247	2.06%	2.06%
19	166	35964.1	222.56	215	3.52%	3.52%
20	126	34510.3	213.56	208	2.67%	2.67%
Minimum					−3.91%	0.14%
Maximum					7.34%	7.34%
Average					0.69%	2.62%

τ′ = 161.6.

1 µCi = 3.7 × 10^4^ Bq.

### Results of conventional quantitative method

The accuracy of rat thyroid ^131^I activity quantification by conventional method is shown in Table 2. Results of deviation for UC-ROI defining method is also listed as a contrast. For the conventional quantitative method, the average for deviations of calculated activity from the gold standard values was 13.51% ± 1.21%. Significant difference of the accuracy can be observed between UC-ROI defining method and conventional method with a paired matched-test (t = 8.461, P < 0.001).

**Table 2:** Accuracy comparation of the two methods.

Rat Number	1	2	3	4	5	6	7	8	9	10
Deviation for UC-ROI defining method	4.86%	7.34%	4.69%	0.33%	3.27%	2.37%	3.91%	3.44%	1.59%	1.48%
Deviation for conventional method	10.85%	12.19%	15.39%	10.67%	20.07%	19.25%	4.62%	15.35%	24.04%	13.21%
Rat Number	11	12	13	14	15	16	17	18	19	20
Deviation for UC-ROI defining method	2.00%	2.59%	3.82%	0.29%	1.61%	0.38%	0.14%	2.06%	3.52%	2.67%
Deviation for conventional method	7.13%	22.71%	4.44%	10.87%	15.44%	12.20%	8.03%	13.60%	12.87%	17.33%

### Results of accuracy with different µ values

**Figure 6:**
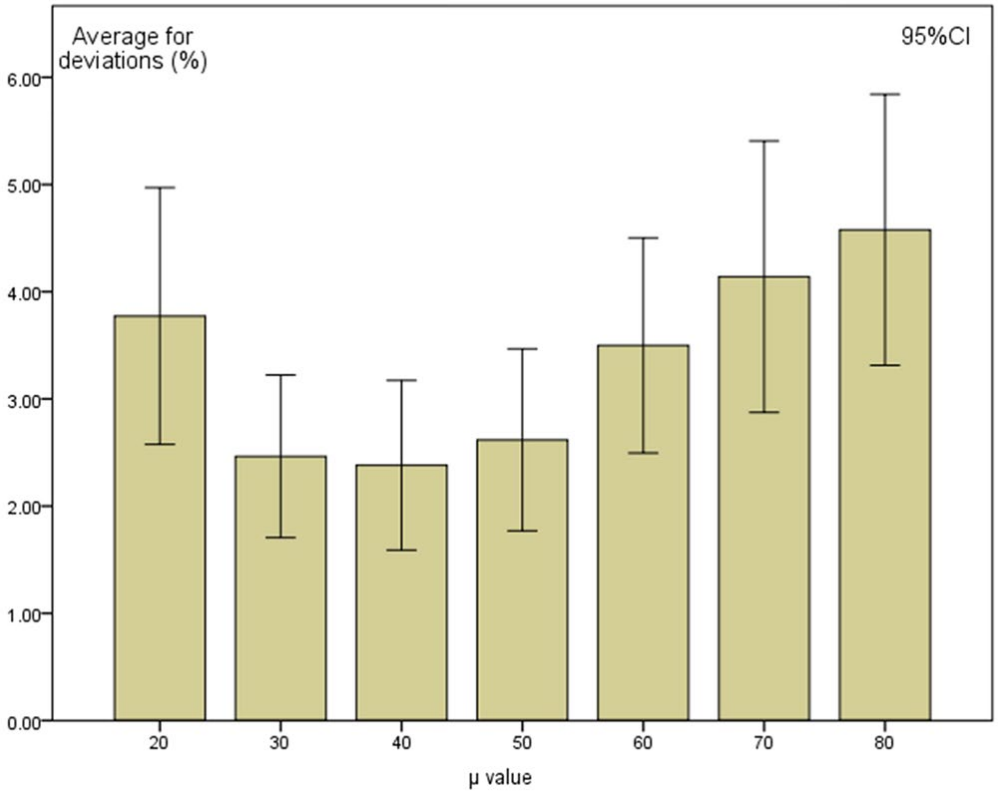
The relationship between averages for deviations and µ values. With different µ values, though subtle differences existing, the accuracy of radioactivity quantification is stable generally. And a better quantitative ability can be observed when µ equals to 30, 40 or 50.

The results above are obtained on the condition that µ equals to 50. We also tested different µ values (µ = 20, 30, 40, 60, 70, and 80), and the averages for deviations of calculated activity from the gold standard values are shown in Fig. 6. The averages of deviations for each µ value are 3.77% ± 0.57%, 2.46% ± 0.36%, 2.38% ± 0.38%, 3.50% ± 0.48%,4.14% ± 0.61% and 4.58% ± 0.60%, respectively.

## Discussion

Thyroid ^131^I radioactive activity quantification is widely applied in clinical practice and research. Radioactive iodine uptake (RAIU) is used to assess the thyroid function^13,14^ and is essential for providing clinical information before ^131^I-therapy in some kinds of thyroid disorders, such as hyperthyroidism^15^ and thyroid carcinoma^16^. After administration of ^131^I, detecting the amount of thyroid uptake radioactive activity will benefit the evaluation of therapeutic process and treatment outcomes. Many animal experiments are based on Iodine-131 so that a promoted method of radioactivity quantification for vivo animal study will be beneficial.

Traditionally, the ^131^I radioactive activity of thyroid uptake is calculated using the equation ((thyroid counts − background counts)/(sensitivity × time))^5,12,13^, where sensitivity is the count rate per unit activity. In our study, we simplified this equation and adopted counts per unit activity (τ) rather than count rate per unit activity. The time factor was neglected because the planar image acquisition for both standard models and rat thyroids was exactly the same, including the parameters such as scanning time (200 s), distance (10 cm), matrix (256 × 256), energy window (364 KeV ± 10%) and others. Considering the scanning time was settled, we used counts per unit activity (τ) directly.

In addition to the factor of scan time, τ can also be influenced by the distance from the radioactive source to the collimator. The value of τ will decrease as the distance grows^5^. Therefore, in order to obtain a quantitative result, a proper distance must be fixed for all the standard models and rat thyroids. In this study, a 10-cm distance was chosen because it was widely used in clinical practice and suitable for all patients^12^. The criterion distance for rat study was not found, so we took clinical practice standard directly.

After the control of systematic scan parameters, the accuracy of quantification is still affected by the target tissue ROI definition and background region selection. The ROI is mainly defined by visual characteristics. However, for the same image, with different ULWL settings, the visual characteristics will change and the ROI selection is influenced. To solve this, our study used a new and easy method for ROI definitions, a UC-ROI defining method based on ULWL and a colour display grade. We proposed a method for setting the ULWL based on the average counts per pixel (α), multiplied by coefficient (µ), as in equations (1) and (2). In this study, we adopt 50 as the µ value. As is shown in Fig. 6, other values will also be reliable. We tested different µ values, and though subtle differences existing, the accuracy of radioactivity quantification remained stable and acceptable generally. The key point is that the value of μ should stay the same for all data processing and ROIs defining. It is more accurate when µ equals to 30, 40, or 50, compared with other conditions.

A promotion for background selection was also taken in this study. The counts for the ROI of the target tissue needed to be corrected by the background to obtain a more reliable result^1,2,10^. However, as explained above, there are random errors with the conventional background correction. Drawing a circular background region is slightly more complex compared with the conventional method, but it is more stable and easy-to-follow.

The five standard models were designed with five different levels of radioactive activity in order to obtain a more exact value of τ. The standard models were covered with 5-mm depth water to imitate the soft tissue overlap of the thyroid of the rat. Note that though the diameter of the plastic model is 60 mm, the ROI selection should be based on visual characteristics rather than the actual model diameter. The values of τ_n_ for each model were 159.6, 161.8, 161.1, 159.8 and 165.7, with very slight differences existing. This was indicated by the UC-ROI defining method and ring-like background selection; the τ is a stable value for the standard models despite the changes in radioactive activity.

After using the τ obtained from standard models to estimate the radioactive activity of the 20 rat thyroids, the results met expectations perfectly. The mean quantification within 5% could be achieved in 19 of 20 rat thyroids, and no significant difference existed between the calculated thyroid ^131^I activity and gold standard values. Our results showed a much better accuracy (0.69% of deviation, and 2.62% of absolute deviation) compared with conventional quantitative method. Both of the two methods are based on visual characteristics. But for the conventional method, ROI was selected on the planar imaging of default ULWL directly and background ROI was selected in an unstable way. Thus in our study, for the conventional method, the average for deviations of calculated activity from the gold standard values was 13.51%, much higher than UC-ROI defining method (2.62%).

In addition to the accuracy, the simplicity and convenience are also the good features of our method. After setting the imaging parameter and image acquisitions of the standard models and target tissues, to quantify the radioactive activity, all we need to do is take another step before selecting the ROI—setting the ULWL. Unlike other studies, the scatter correction^7^ and partial volume effect correction^8^ are not necessary with our method. The ROI borders are chosen based on visual characteristics according to the UC-ROI defining method, so the CT component is not needed for obtaining the boundary. The attenuation can be simulated by the depth of water in the standard models. We used a 5-mm depth water for simulating the rat thyroid attenuation.

The UC-ROI method can be widely used in animal experiments. With a τ obtained from standard models, it is convenient to calculate the radioactive activity of thyroid uptake for animals after ^131^I treatment. In addition to the thyroid, ^131^I uptake of other organs (the liver, the bladder, stomach and etc.) can also be quantified by the UC-ROI method in the vivo rat study if a ROI border can be defined clearly. For instance, if a sort of liver targeted radiolabeled agent was applied in rat experiments, the UC-ROI defining method can be adopt for planar imaging quantification theoretically.

## Limitations

This promoted method is one that will work best in animal studies, especially for these with minimum background. However, while the background increasing, the border of the target tissue will be mixed with the background and become undefined. In other words, this UC-ROI defining method will be inaccuracy in the conditions with high background.

## Conclusion

We report an accurate and simple method for thyroid uptake ^131^I radioactive activity quantification by gamma-camera planar imaging *in vivo* rat study with a high-energy collimator of a commercial SPECT and image processing workstation by a UC-ROI defining method. The long-term goal is to verify the accuracy in other organs (like the liver, the bladder, stomach and etc.) and investigate more applications of this ROI and background defining method in animal experiments.
